# Clinical characteristics and risk factors of in-hospital gastrointestinal bleeding in patients with acute myocardial infarction

**DOI:** 10.3389/fcvm.2022.933597

**Published:** 2022-09-27

**Authors:** Liang Zhong, Xingpu Quan, Peizhu Dang, Manyun Tang, Hang Yu, Fengwei Guo

**Affiliations:** ^1^Department of Cardiovascular Surgery, The First Affiliated Hospital of Xi'an Jiaotong University, Xi'an, China; ^2^Department of Gastroenterology, The First Affiliated Hospital of Xi'an Jiaotong University, Xi'an, China; ^3^Department of Cardiovascular Medicine, The First Affiliated Hospital of Xi'an Jiaotong University, Xi'an, China

**Keywords:** acute myocardial infarction, heart failure, gastrointestinal bleeding, risk stratification, propensity score matching

## Abstract

**Background:**

Gastrointestinal bleeding (GIB) is one of the most serious complications of acute myocardial infarction (AMI) and is correlated with poor outcomes.

**Objective:**

To evaluate the prevalence, risk factors and in-hospital mortality of GIB in patients with AMI.

**Methods:**

This observational case-control study retrospectively enrolled consecutive patients with AMI from the Department of Cardiovascular Medicine and Cardiovascular Surgery of the First Affiliated Hospital of Xi'an Jiaotong University from January 2015 to December 2020. GIB after AMI was identified by International Classification of Diseases (ICD) codes from inpatient medical settings and validated by medical record review. AMI patients without GIB were accordingly classified as the control group. Propensity score matching (PSM) was used to match with the GIB group and the control group. All anonymized clinical data were provided by the Biobank of the First Affiliated Hospital of Xi'an Jiaotong University.

**Results:**

A total of 5,868 AMI patients were enrolled, 0.87% (51/5,868) of whom developed GIB after AMI. On the univariate analysis, history of diabetes, chronic kidney disease, Killip IV, a lower hemoglobin concentration, a higher serum level of creatinine, blood urea nitrogen and D-dimer were closely associated with the risk of GIB (*P* < 0.05). On the multivariable analysis, a lower hemoglobin concentration (OR: 0.93, 95% CI: 0.89–0.96, *P* < 0.001) was independently associated with the risk of GIB. Patients with GIB had a much higher in-hospital mortality rate than those without GIB (14.3 vs. 2.1%, *P* = 0.047). In-hospital mortality among patients with GIB after AMI appeared to be associated with a decreased hemoglobin concentration (OR: 0.93, 95% CI: 0.86–0.99, *P* = 0.045) and Killip IV (OR: 51.59, 95% CI: 2.65–1,005.30, *P* = 0.009).

**Conclusion:**

The history of diabetes, poor renal function and heart failure were associated with the high risk of GIB in patients experiencing AMI. The in-hospital mortality in patients with AMI complicating GIB was higher than that in patients without GIB and was associated with a decreased hemoglobin concentration and high Killip classification.

## Introduction

Acute myocardial infarction (AMI) is a serious increasing global health problem due to its high mortality and morbidity rates (1). The emphasis of treatment for AMI is prompt myocardial reperfusion, including the application of thrombolytic therapy or primary percutaneous coronary intervention (PCI) (2). Antithrombotic (anticoagulant or antiplatelet) effects are important mechanisms of PCI, which can reduce even prevent perioperative and long-term ischemic cardiovascular events, including stent thrombosis and recurrent myocardial infarction (3, 4). Substantial progress has been made in myocardial infarction treatment, such as early reperfusion therapy, which has extensively decreased the mortality rate in patients with AMI (5).

However, bleeding is one of the most serious complications of AMI which directly associated with an increased mortality (6). Gastrointestinal bleeding (GIB) is a joint adverse drug reaction that occurs in patients receiving dual antiplatelet medication, with an incidence of 5% to more than 10% (7). Bleeding events after PCI are related to increasement of short- and long-term morbidity and mortality (8, 9). GIB can affect the prognosis of AMI patients and increase the risk of major adverse cardiac events (MACEs) in the early stage (during hospitalization and within 30 days after discharge) and late stage (10–12). GIB is associated with markedly increased mortality and morbidity and can be life-threatening in patients with acute coronary syndromes (ACSs) (13). However, there is no systematic report on the risk factors and prognosis of GIB in AMI patients in China. Here, we conducted a real-world study to evaluate the risk factors and in-hospital outcomes of GIB in AMI patients in a Chinese population.

## Methods

### Study design

This was an observational, case-control study. Anonymized clinical data were collected from the Biobank of the First Affiliated Hospital of Xi'an Jiaotong University. The Ethics Committee of the First Affiliated Hospital of Xi'an Jiaotong University approved this study (no. XJTU1AF2021LSK116), and informed consent was obtained. All methods were carried out in accordance with relevant guidelines and regulations and the principles of the Declaration of Helsinki.

### Participants

A total of 5,868 consecutive patients hospitalized for the first time for AMI were enrolled between January 2015 and December 2020 at the First Affiliated Hospital of Xi'an Jiaotong University (Shaanxi, China). AMI was defined based on the universal definition criteria established by the American College of Cardiology (14). GIB was defined as clinical events of bleeding (coffee ground emesis, hematemesis, melena, or hematochezia) diagnosed by a physician or the presence of blood in the upper or lower gastrointestinal tract on endoscopic evaluation (15).

The inclusion criterion was a diagnosis of AMI, including non-ST-segment elevation myocardial infarction (NSTEMI) and ST-segment elevation myocardial infarction (STEMI). The exclusion criteria were prior history of bleeding within 1 month and baseline data missing.

### Data collection

Collected data included demographic characteristics, medical history, clinical information, laboratory results, and oral medications within 24 h after admission. Venous blood was analyzed in the Core Laboratory of the First Affiliated Hospital of Xi'an Jiaotong University for examinations of blood biochemistry, hemoglobin, blood urea nitrogen (BUN), serum creatinine, and D-dimer. Coronary angiography data were also collected and the angiographic burden of AMI patients was quantified by the modified Gensini Score (16).

### Propensity score matching

A propensity score-matching (PSM) analysis adjusted to sex, age, myocardial infarction type (including STEMI and NSTEMI), and hospital stay was performed in order to reduce bias. The subjects were matched in a 1:1 ratio and the caliper value was 0.002. Through the application of PSM, the participants of the GIB group and the control group were paired for the factors mentioned above. This statistical approach reduced the possibility of introducing confounding factors by providing a balanced distribution of selected characteristics of the two groups.

### Statistical analysis

Statistical analysis was performed using SPSS version 26.0 (SPSS Inc., Chicago, IL, USA). Mean values with standard deviations (SDs) and counts with percentages were used to describe clinical characteristics and factors related to GIB. Differences were evaluated with a paired *T*-test or Wilcoxon test for continuous variables and a chi-square test or Fisher's exact test for categorical variables. Univariate and multivariate logistic regressions were used to determine possible factors influencing the prevalence and in-hospital outcomes of GIB. Odds ratios (ORs) and 95% confidence intervals (CIs) were calculated. All *P*-values were two-sided, and *P* < 0.05 was considered statistically significant.

## Results

**Figure 1:**
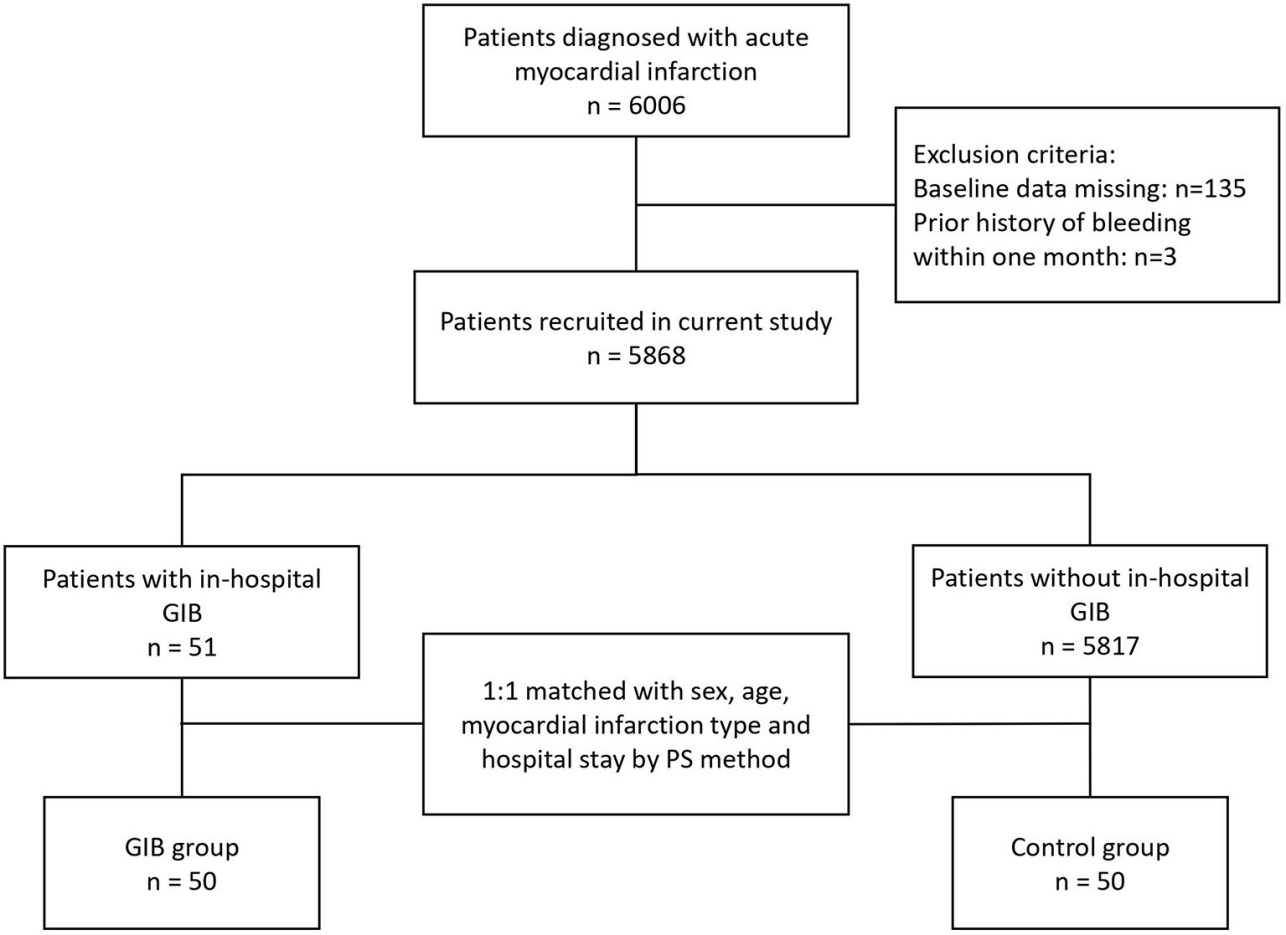
Flowchart of the selection process of the current study. AMI, acute myocardial infarction; GIB, gastrointestinal bleeding.

A total of 5,868 AMI patients were enrolled, and the occurrence of in-hospital GIB was clinically confirmed in 51 patients (51/5868, 0.87%). Before PSM, 80.5% were males. Among the GIB patients, 22/51 (43.1%) had STEMI, and 29/51 (56.9%) had NSTEMI. After PSM, 50 patients who developed in-hospital GIB were finally assigned to the GIB group and 50 patients pair matched with sex, age, myocardial infarction type and hospitalization time were assigned to the control group (Figure 1).

### Population comparison before and after PSM

The original demographics and characteristics of all enrolled patients are shown in Table 1. Before PSM, the average age in the GIB group was significantly higher than that in the control group (67.67 ± 10.35 vs. 61.49 ± 12.15, *P* < 0.001), and the mean hospitalization time in the GIB group was significantly longer than that in the control group (10.53 ± 12.07 vs. 5.69 ± 5.15, *P* < 0.001). Moreover, there were more STEMI patients in the GIB group (43.1 vs. 28.5%, *P* < 0.05). Then, after PSM, all matched factors were well-balanced and comparable between the two groups, and there were no significant differences in sex, age, myocardial infarction type and mean hospitalization time (Table 1).

**Table 1:** Clinical characteristics of in-hospital GIB in AMI patients before and after PSM.

**Characteristics**	**Before PSM (*****N*** = **5,868)**				**After PSM (*****N*** = **100)**			
	**GIB group**	**Control group**	**SMD**	***P*-value**	**GIB group**	**Control group**	**SMD**	***P*-value**
	**(*N* = 51)**	**(*N* = 5,817)**			**(*N* = 50)**	**(*N* = 50)**		
Male (*n*, %)	44 (86.3)	4,677 (80.4)	0.158	0.381	43 (86.0)	41 (82.0)	0.109	0.785
Age (years)	67.67 ± 10.35	61.49 ± 12.15	0.547	<0.001	67.46 ± 10.35	63.36 ± 12.65	0.355	0.079
Myocardial infarction type (*n*, %)			0.310	0.031			0.206	0.412
STEMI	22 (43.1)	1,655 (28.5)			22 (44.0)	17 (34.0)		
NSTEMI	29 (56.9)	4,162 (71.5)			28 (56.0)	33 (66.0)		
Mean hospitalization time (days)	10.53 ± 12.07	5.69 ± 5.15	0.522	<0.001	9.84 ± 11.13	6.86 ± 9.07	0.294	0.145

GIB, gastrointestinal bleeding; AMI, acute myocardial infarction; PSM, propensity score matching; SMD, standardized mean difference; STEMI, ST-elevation myocardial infarction; NSTEMI, non-ST-segment elevation myocardial infarction.

### Baseline characteristics

Table 2 showed the differences in demographic and clinical characteristics of AMI patients with and without in-hospital GIB after PSM. Compared with patients without in-hospital GIB, patients who developed in-hospital GIB were more likely to have a much lower hemoglobin count (99.38 ± 24.06 vs. 129.64 ± 15.82, *P* < 0.001), and a higher serum level of D-dimer (3.31 ± 6.78 vs. 1.47 ± 2.65, *P* = 0.001), BUN (8.36 ± 6.05 vs. 5.58 ± 3.91, *P* = 0.028) and creatinine (116.69 ± 130.51 vs. 80.60 ± 56.34, *P* = 0.038). Moreover, history of diabetes, Killip level IV and chronic kidney disease (CKD) were also more common in the GIB group (*P* < 0.05).

**Table 2:** Differences in demographic and clinical characteristics of AMI patients with and without in-hospital GIB.

**Patients**	**In total**	**GIB group**	**Control group**	***P*-value**
	**(*N* = 100)**	**(*N* = 50)**	**(*N* = 50)**	
Smoker (*n*, %)	51 (56.7)	22 (53.7)	29 (59.2)	0.598
Drinker (*n*, %)	9 (9.2)	5 (10.2)	4 (8.2)	1.000
SBP (mmHg)	126.09 ± 27.27	127.24 ± 27.86	125.00 ± 26.95	0.432
DBP (mmHg)	77.61 ± 15.55	75.20 ± 15.18	79.88 ± 15.71	0.301
**Blood biochemistry**
Hb (g/L)	114.51 ± 25.33	99.38 ± 24.06	129.64 ± 15.82	< 0.001
WBC (×10^9^/L)	8.08 ± 3.45	8.16 ± 3.25	8.00 ± 3.67	0.721
Platelet (×10^9^/L)	212.55 ± 106.99	214.36 ± 123.28	210.74 ± 89.01	0.567
BUN (mmol/L)	6.94 ± 5.23	8.36 ± 6.05	5.58 ± 3.91	0.028
Creatinine (μmol/L)	98.28 ± 100.92	116.69 ± 130.51	80.60 ± 56.34	0.038
D-dimer (mg/L)	2.31 ± 5.03	3.31 ± 6.78	1.47 ± 2.65	0.001
**Medications**
Aspirin (*n*, %)	79 (88.8)	34 (82.9)	45 (93.8)	0.177
P2Y12 inhibitors (*n*, %)	83 (93.3)	36 (87.8)	47 (97.9)	0.091
Anticoagulant (*n*, %)	36 (40.4)	11 (26.8)	25 (52.1)	0.016
PPI (*n*, %)	83 (93.3)	38 (92.7)	45 (93.8)	1.000
PCI (*n*, %)	66 (73.3)	23 (56.1)	43 (87.8)	0.001
**Killip classification (** ***n*** **, %)**
I	62 (66.7)	25 (58.1)	37 (74.0)	0.115
II	17 (18.3)	8 (18.6)	9 (18.0)	1.000
III	5 (5.4)	3 (7.0)	2 (4.0)	1.000
IV	7 (7.5)	6 (14.0)	1 (2.0)	0.031
**Comorbidities**
Hypertension (*n*, %)	48 (92.0)	23 (54.8)	25 (50.0)	0.649
Diabetes (*n*, %)	25 (25.0)	18 (36.0)	7 (14.0)	0.002
Stroke (*n*, %)	13 (14.1)	7 (16.7)	6 (12.0)	0.522
CKD (*n*, %)	9 (9.0)	8 (16.0)	1 (2.0)	0.010
Tumor (*n*, %)	9 (9.0)	5 (10.0)	4 (8.0)	0.727
Gensini score	62.50 ± 36.53	69.31 ± 42.25	58.48 ± 32.54	0.747
In-hospital death (*n*, %)	7 (7.8)	6 (14.3)	1 (2.1)	0.047

AMI, acute myocardial infarction; GIB, gastrointestinal bleeding; SBP, systolic blood pressure; DBP, diastolic blood pressure; Hb, hemoglobin; WBC, white blood cell; BUN, blood urea nitrogen; PPI, proton pump inhibitors; PCI, percutaneous coronary intervention; CKD, chronic kidney disease.

### Risk factors of AMI patients with in-hospital GIB

**Figure 2:**
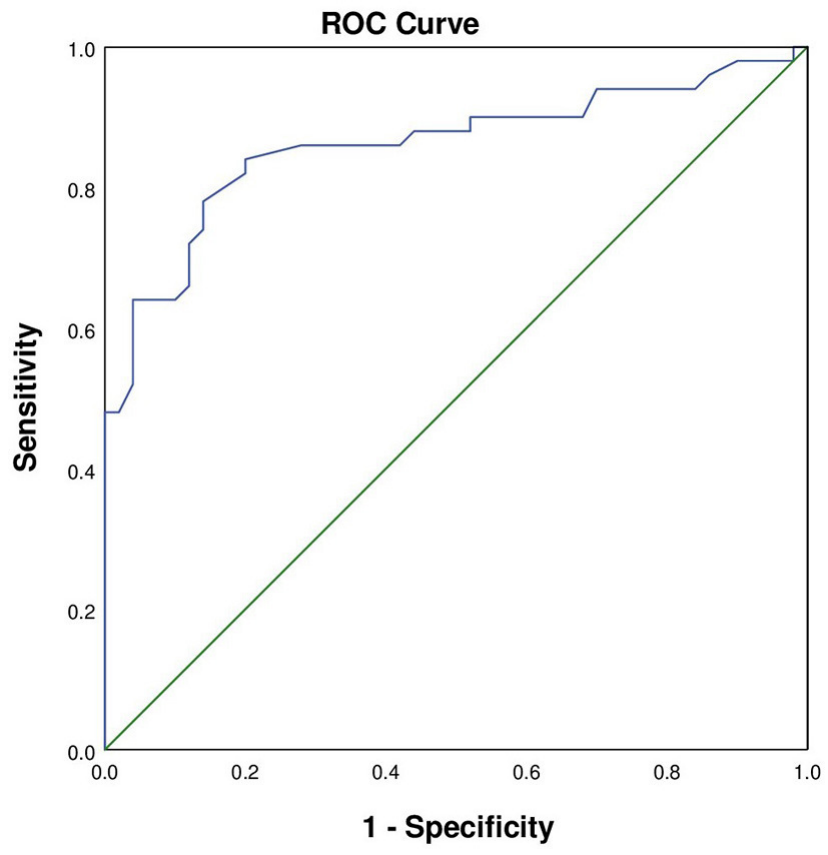
The ROC curve of AMI patients with in-hospital GIB. The area under the curve was 0.860 (95% CI: 0.78–0.94, *P* = 0.039). The sensitivity was 0.84 and the specificity was 0.80.

According to the analysis of differences in demographic and clinical characteristics between the GIB group and the control group, factors used to predict in-hospital GIB were identified as hemoglobin concentration, serum level of creatinine, BUN, D-dimer, and history of diabetes and Killip IV. All these factors were then assessed by multivariable regression analysis, and lower hemoglobin concentration was found to be an independent risk factor for in-hospital GIB following AMI (OR: 0.92, 95% CI: 0.89–0.96, *P* < 0.001; Table 3). The receiver operating characteristic (ROC) curve was plotted with hemoglobin concentration. The area under the curve was 0.86 (95% CI: 0.78–0.94, *P* = 0.039) with a sensitivity of 0.84 and a specificity of 0.80, indicating that hemoglobin had a high discriminative ability for in-hospital GIB after AMI (Figure 2).

**Table 3:** Logistic regression analysis of AMI patients with in-hospital GIB.

**Characteristics**	**OR**	**95% CI**	***P*-value**
Hb	0.92	0.89–0.96	< 0.001
Creatinine	1.00	0.99–1.01	0.453
BUN	0.99	0.83–1.17	0.877
D-dimer	1.08	0.96–1.22	0.212
Diabetes	4.10	0.92–18.15	0.063
Killip IV	12.57	0.62–255.99	0.100

OR, odd ratio; CI, confidence interval. Other abbreviations as in Table 2.

### Factors affecting in-hospital outcomes in AMI patients with in-hospital GIB

The in-hospital outcome of interest was all-cause death related or not related to in-hospital bleeding. Univariate and multivariate analyses were performed to evaluate the risk factors of outcomes, and the results are shown in Table 4. On the univariate analysis, a lower hemoglobin concentration (OR: 0.95, 95% CI: 0.90–1.00, *P* = 0.049), a higher BUN concentration (OR: 1.16, 95% CI: 1.01–1.33, *P* = 0.030) and Killip IV (OR: 13.67, 95% CI: 1.88–99.35, *P* = 0.010) were associated with the in-hospital death of AMI patients with GIB. After adjusted for confounding factors on the multivariable analysis, a lower hemoglobin concentration (OR: 0.93, 95% CI: 0.86–0.99, *P* = 0.045) and Killip IV (OR: 51.59, 95% CI: 2.65–1,005.30, *P* = 0.009) were found to independently associate with the in-hospital death of AMI patients with GIB.

**Table 4:** Factors affecting in-hospital outcomes in AMI patients with in-hospital GIB.

**Characteristics**	**Univariate model**		**Multivariate model**	
	**OR (95% CI)**	***P*-value**	**OR (95% CI)**	***P*-value**
Age	1.04 (0.96–1.14)	0.352		
Hb	0.95 (0.90–1.00)	0.049	0.93 (0.86–0.99)	0.045
WBC	1.14 (0.89–1.47)	0.311		
Platelet	0.99 (0.98–1.00)	0.170		
BUN	1.16 (1.01–1.33)	0.030	1.08 (0.91–1.29)	0.372
Creatinine	1.00 (0.99–1.01)	0.741		
D-dimer	1.00 (0.86–1.16)	0.973		
Diabetes	4.29 (0.70–26.24)	0.115		
Killip IV	13.67 (1.88–99.35)	0.010	51.59 (2.65–1,005.301)	0.009
Hypertension	1.20 (0.22–6.61)	0.834		

Abbreviations as in Tables 2, 3.

## Discussion

In this single center, retrospective analysis study, patients with AMI complicated with GIB were enrolled and analyzed. The independent risk factors for GIB in AMI patients during hospitalization included history of diabetes, heart failure (Killip IV) and poor renal function. Moreover, AMI patients with GIB had an increased risk of death compared to AMI patients without GIB, which was associated with a lower hemoglobin concentration and heart failure (Killip IV).

The incidence and mortality rates of GIB after AMI have progressively declined over the past decades (17). The patients enrolled in this study were from one of the most prestigious medical centers in Western China. In this study, among the AMI patients, the GIB prevalence was 0.87%, which was lower than that in previous investigations ranging from 1.1 to 3.0% (18). In the ACUITY (Acute Catheterization and Urgent Intervention Triage Strategy) trial, GIB served as the second major common source of non-coronary artery bypass graft (CABG)-related bleeding in the entire study population, which ranked only after access site bleeding (8). A temporal trend study for GIB indicated that despite aggressive treatment for ACS, the incidence of GIB associated with PCI decreased over a decade, which may explain the results in our research (19). In addition, Chinese physicians tend to prevent GIB by applying proton-pump inhibitors, which could also explain the reduced incidence of GIB.

Risk factors of gastrointestinal hemorrhage after AMI, such as older age, history of diabetes, high Killip classification and chronic renal insufficiency, have been documented (20–22). Our results demonstrated that the occurrence of GIB in patients with AMI was closely associated with history of diabetes, high Killip classification and chronic renal insufficiency. Moscucci et al. (23) conducted a large observational study in patients with AMI enrolled in the GRACE (Global Acute Coronary Events Registry) and observed that older age, female sex, history of bleeding, and renal insufficiency were independent predictors of major bleeding Sarajlic et al. (24) also found that blood glucose, smoking status, and previous GIB were predictors of major bleeding among 149,447 patients with AMI enrolled in the SWEDEHEART (Swedish Web-system for Enhancement and Development of Evidence-based care in Heart disease Evaluated According to Recommended Therapies) registry. Although most of these factors are immutable, their recognition allows for better risk stratification and more active management to reduce associated morbidity and mortality.

The mechanisms of in-hospital bleeding in patients with AMI are multifactorial. It has been assumed that the presence of local vascular changes is a common cause of the increased incidence of bleeding complications in elderly and diabetic patients (25). In addition, insufficient tissue perfusion adversely affects the coagulation system and platelet function, which may lead to gastritis or ulcers that increase the risk of upper gastrointestinal bleeding (23). Moreover, the “over-administration” effect, which results in high blood concentration, is the main mechanism of the increased risk of bleeding due to renal insufficiency (26). To reduce the risk of bleeding, all antithrombotic and antiplatelet drug agents should be administered in combination with renal function (27).

The outcomes of patients presenting with AMI have improved over time due to improvements in systems of care (e.g., symptom recognition, door-to-balloon time, etc.), advances in primary PCI techniques and their widespread adoption (28). However, in previous studies, gastrointestinal hemorrhage in AMI patients during hospitalization have been shown to be associated with increased short-and long-term mortality (17, 29). Our results supported this opinion. Patients with in-hospital GIB had a much higher in-hospital mortality rate than those without (2.1 vs. 14.3%). GIB complicating AMI leads to increased mortality, but there're still many unknowns about what and how factors affect the in-hospital outcomes of GIB. We sought to examine clinical and procedural factors associated with GIB. Compared with the survivors, patients who died in the hospital had a lower level of hemoglobin, a higher level of BUN, and a higher Killip classification.

Eikelboom et al. (30) found that the severity of GIB was associated with increased mortality. The mechanisms linking GIB with mortality are probably multifactorial. Massive GIB can lead to hemodynamic compromise, which results in death. While mild to moderate GIB can cause systemic inflammation with a prothrombic state, which in turn may lead to recurrent ischemic events. In addition to these direct effects, some indirect effects also affect the prognosis of AMI patients. For example, blood transfusion may increase oxidative stress and lead to a paradoxical decrease in oxygen delivery, all of which could contribute to worse outcomes (31). Moreover, even mild bleeding that does not require transfusion may lead to discontinuation of antithrombotic therapy, which indirectly affects prognosis.

Due to the widespread application of PCI and its clinical benefits over thrombolytic therapy, the majority of AMI patients admitted to our hospital have undergone PCI, excluding those who had economic difficulties. In this study, the use of anticoagulants and PCI treatment rates were significantly lower in patients with AMI combined GIB than those in the control group. This was due to the higher PCI risk and contraindications of anticoagulant therapy in AMI patients with GIB, which led to a statistical bias in this study.

There are some limitations that need to be noted. First, this was a single-center, retrospective study, and the cohort of patients was not large enough. A relatively small number of female AMI patients were enrolled in the present study. Second, the sample size in this study was relatively small because of the low incidence of GIB. Follow-up research based on a larger cohort is warranted to further explore the prognosis of and preventive factors associated with GIB.

## Conclusion

In summary, in this single center, retrospective analysis, we identified that diabetes, heart failure (Killip IV) and poor renal function were associated with the risk of GIB. Moreover, AMI patients with GIB had a higher risk of all-cause death during hospitalization and a decreased hemoglobin concentration and heart failure were found to contribute to this increased mortality. The present study provides new evidence, allowing a better understanding of GIB and providing guidance for its clinical management.

## Data availability statement

The raw data supporting the conclusions of this article will be made available by the authors, without undue reservation.

## Ethics statement

The studies involving human participants were reviewed and approved by the Ethics Committee of the First Affiliated Hospital of Xi'an Jiaotong University (No. XJTU1AF2021LSK116). The patients/participants provided their written informed consent to participate in this study.

## Author contributions

FG and HY designed and supervised the study. LZ, XQ, and MT wrote and revised the paper. PD collected the clinical data, obtained informed consent from the patients, and analyzed the data. All authors contributed to the article and approved the final version for submission.

## Conflict of interest

The authors declare that the research was conducted in the absence of any commercial or financial relationships that could be construed as a potential conflict of interest.

## Publisher's note

All claims expressed in this article are solely those of the authors and do not necessarily represent those of their affiliated organizations, or those of the publisher, the editors and the reviewers. Any product that may be evaluated in this article, or claim that may be made by its manufacturer, is not guaranteed or endorsed by the publisher.
